# Supplementary material for: A lipid gating mechanism for the channel-forming O antigen ABC transporter
Source: Nat Commun. 2019 Feb 18;10:824. doi: 10.1038/s41467-019-08646-8 (PMC6379404; doi:10.1038/s41467-019-08646-8)
Supplement: Supplementary file 1 — Supplementary Information [file 41467_2019_8646_MOESM1_ESM.pdf]

A lipid gating mechanism for the channel-forming O antigen ABC transporter

Christopher A. Caffalette[1], Robin A. Corey[2], Mark S. P. Sansom[2], Phillip J. Stansfeld[2], and Jochen Zimmer[1]

[1] Molecular Physiology and Biological Physics, University of Virginia School of Medicine, Charlottesville, VA 22908, USA, [2] Department of Biochemistry, University of Oxford, OX1 3QU, UK

* Corresponding author: jochen_zimmer@virginia.edu

Supplementary Information

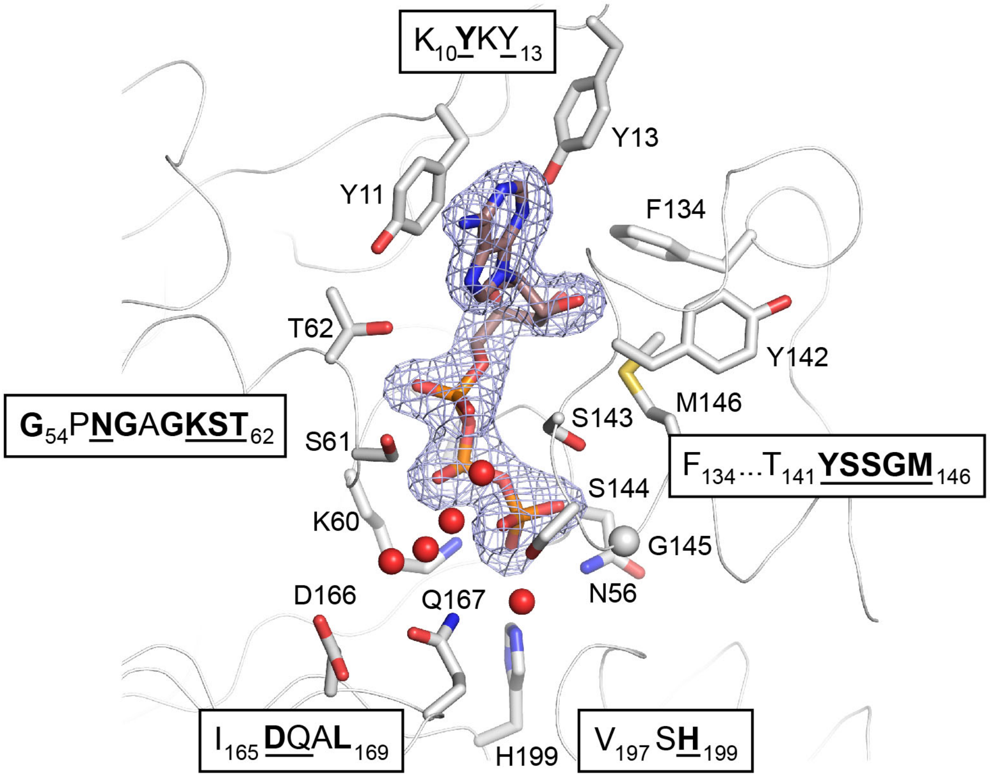

Supplementary Figure 1| Coordination of ATP at the Wzt dimer interface. The Wzt NBDs are shown as a ribbon with side chains and water molecules contacting ATP shown as sticks and red spheres, respectively. ATP-coordinating sequence motifs are shown as a text box with conserved residues in bold, residues shown in the image are underlined. ATP is shown as sticks together with an unbiased SigmaA-weighted Fo-Fc difference electron density contoured at 4.5σ (blue mesh).

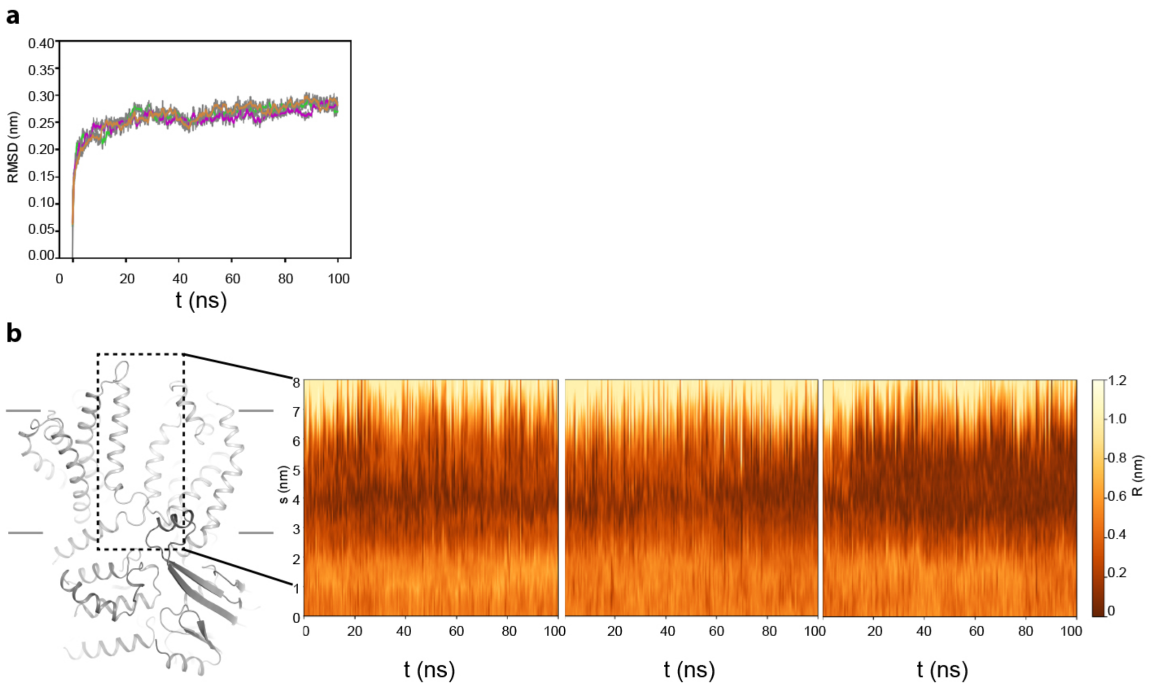

Supplementary Figure 2| Channel dimensions of the ATP-bound WzmWzt transporter. (**a**) RMSD of protein C-alpha carbons from three atomistic 100 ns molecular dynamics simulations of ATP-bound AaWzmWzt$_{EQ}$ in a POPE lipid bilayer. (**b**) Channel dimensions over each of the 100 ns simulations, as measured using the CHAP package[27,28]. The Y-axis, s, represents the position along the channel axis as indicated by the dashed box. R: Channel radius at position s.

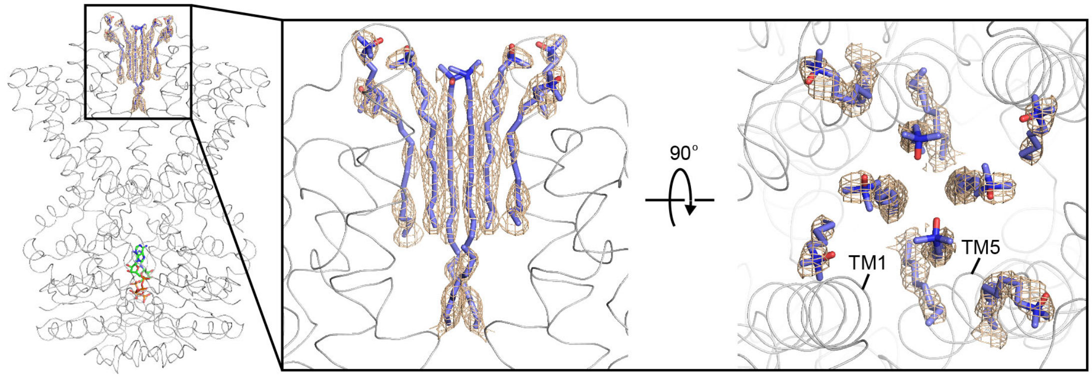

Supplementary Figure 3| Detergent molecules plug the periplasmic channel exit. Unbiased SigmaA weighted Fo-Fc difference electron density contoured at 2.5σ for six full-length and two partial LDAO molecules within the periplasmic channel opening (wheat mesh). LDAO molecules are shown as red and blue sticks, ATP is shown as colored sticks, and WzmWzt is shown as a ribbon.

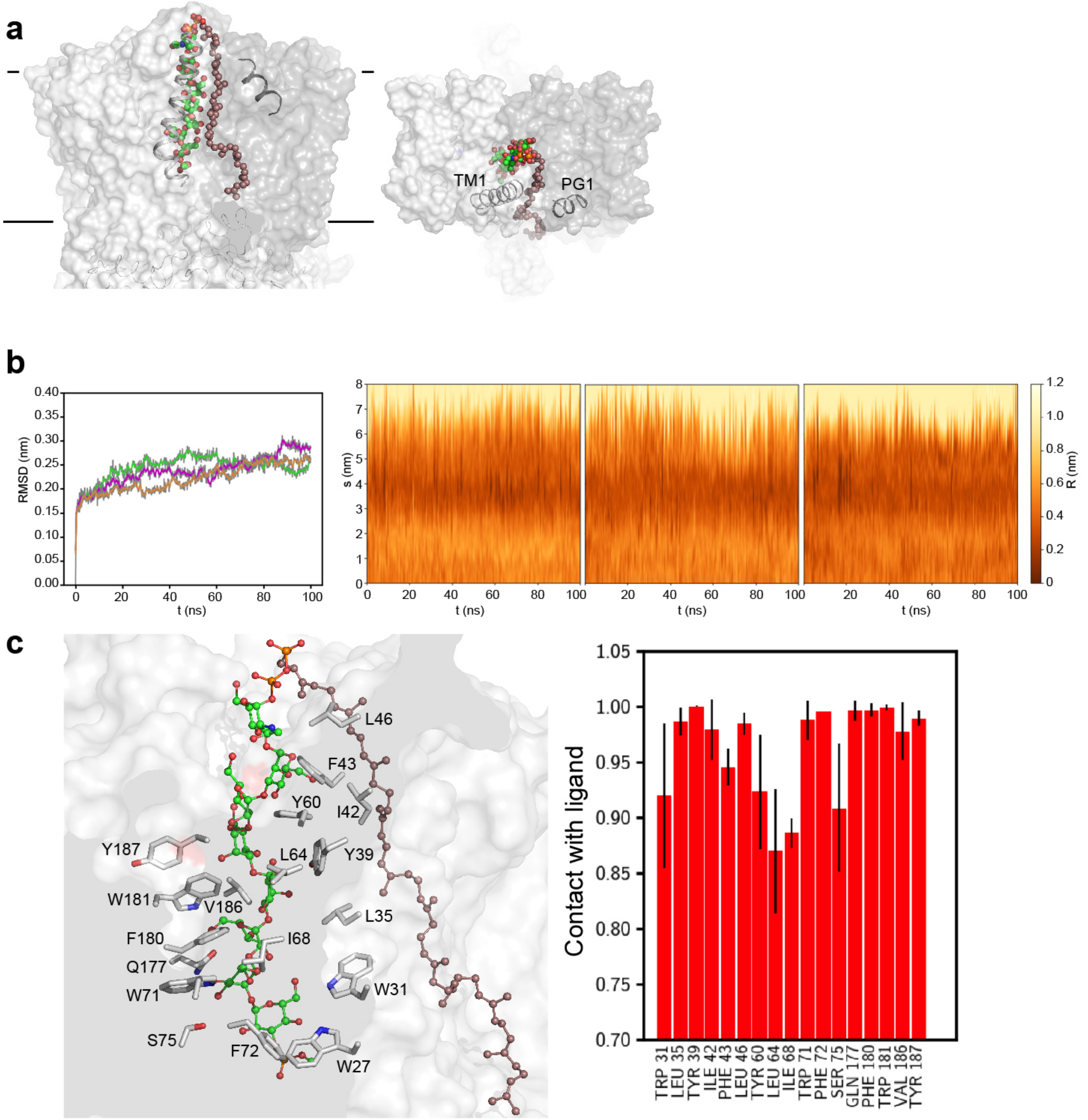

Supplementary Figure 4| MD analysis of substrate-bound WzmWzt. (**a**) Side and periplasmic views of the modeled AaWzmWzt$_{EQ}$ transporter bound to an E. coli O9a antigen repeat unit with a methylphosphate derivatized non-reducing end after a 100 ns all atom MD simulation. The oligosaccharide is shown as spheres in green for carbon atoms. The undecaprenyl moiety is shown as violet beads. TM helix 1 and the periplasmic gate helix (PG1) are shown as cartoons. (**b**) RMSD and pore dimensions of the O9a-bound Wzm channel in three simulations, as per Supplementary Fig. 2. (**c**) Left: Coordination of the O9a antigen inside the Wzm channel. Wzm is shown as a clipped surface with selected oligosaccharide-coordinating residues shown as sticks. Residues are labeled for one protomer only. Right: Contact analysis representing interactions between channel-lining residues and the O9a oligosaccharide during the 100 ns simulations for three simulations. Contact is defined as inter-residue distances of less than 4.5 Å and is expressed as the mean normalized % interaction with error bars showing one standard deviation.

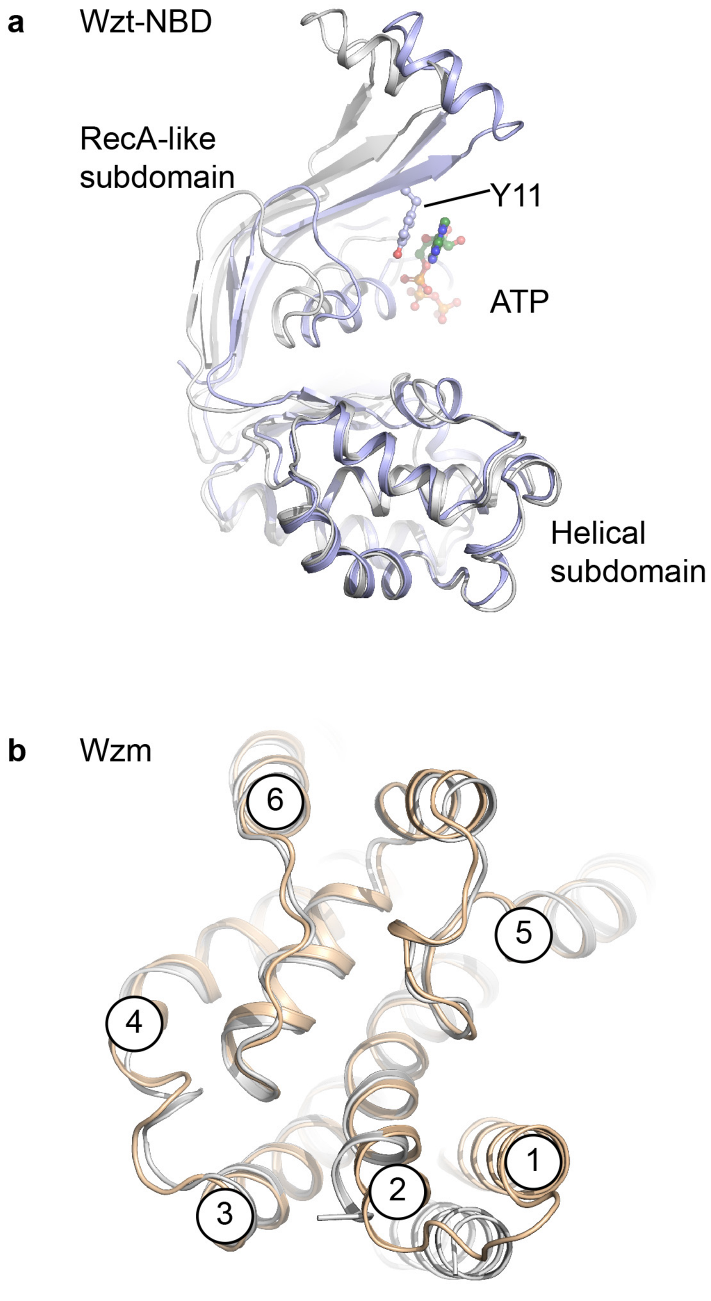

Supplementary Figure 5| Individual comparison of the Wzt and Wzm structures in the ATP-bound and nucleotide-free states. The domains were individually aligned based on the helical subdomain (Wzt) (**a**) or TM helices 2-6 (Wzm) (**b**) by Secondary Structure Matching (SSM) in Coot[52] and visualized as cartoon representations. The nucleotide-free state is colored gray and ATP is shown as 'ball-and-sticks'.

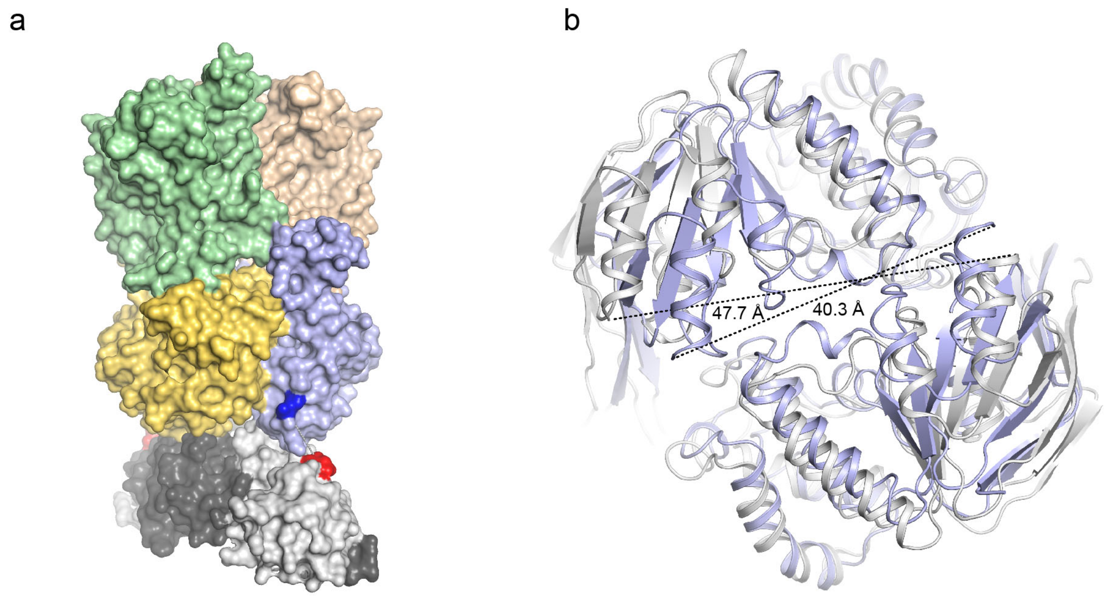

Supplementary Figure 6| Nucleotide-induced movement of the Wzt-NBD C termini. (**a**) Localization of the C-terminal carbohydrate-binding domains (CBDs) relative to the truncated AaWzmWzt transporter. The CBD was manually docked to form the WzmWzt-CBD complex using CBD coordinates PDB:5HNO. The CBD dimer is shown in light and dark gray. The Wzt C-terminal residue is highlighted in blue, and the CBD dimer N-terminal residues are highlighted in red. (**b**) Cytosolic view of the ATP-bound and nucleotide-free AaWzmWzt transporter structures shown in blue and gray, respectively.
