# Supplementary material for: A lipid gating mechanism for the channel-forming O antigen ABC transporter
Source: Nat Commun. 2019 Feb 18;10:824. doi: 10.1038/s41467-019-08646-8 (PMC6379404; doi:10.1038/s41467-019-08646-8)
Supplement: Supplementary file 3 — Description of Additional Supplementary Files [file 41467_2019_8646_MOESM3_ESM.docx]

**Description of Supplementary Files**

**File Name:** Supplementary Movie 1.

**Description:** Movie showing the replacement of the bound oligosaccharide with the lipid plug. Shown are frames between 1.8 and 2.4 μs, where each frame is 50 ns. Colors and representations as per Fig. 4a.The remaining lipids and solvent molecules are removed for clarity.

**File Name:** Supplementary Movie 2.

**Description:** Nucleotide induced conformational changes of AaWzmWztEQ. Shown is a morph from the nucleotide-free to ATP-bound conformation of AaWzmWztEQ in different orientations. Each view of the transporter shows two open to ATP-bound cycles. The transporter is shown as a cartoon representation and semi-transparent surface.
